# Framework for evaluation of food safety in the circular food system

**DOI:** 10.1038/s41538-024-00276-9

**Published:** 2024-06-19

**Authors:** H. J. van der Fels-Klerx, E. D. van Asselt, B. Berendsen, M. F. Focker

**Affiliations:** Wageningen Food Safety Research, Wageningen, Netherlands

**Keywords:** Chemical safety, Microbiology

## Abstract

In order to minimise food waste, side streams from feed and food production are increasingly being (re-) used in food supply chains. Such reuse contributes to the desire to implement circularity in food and agricultural systems. However, the reuse of side products in circular food systems may impact food safety, for instance, contaminant residues present at low levels in biomass may accumulate when reusing streams. In order to assess potential food safety issues related to circular food systems, a framework has been developed in this study. Based on this framework, appropriate actions can be taken to prevent from human health risks. The framework consists of three steps: 1. Describing the changes in the food supply chain as a result of the circularity transition; 2. Identifying potential food safety hazards related to the change; and 3. Prioritising food safety hazards related to the circularity transition. For the prioritisation, both the presence of the hazards in final foods and the effects of the hazards on human health need to be assessed. Persistence of the hazard in the environment and potential transfer from the environment to the final food product are relevant elements to include. The framework was tested in three case studies, showing that it allows for a prioritisation between hazards. Based on the case study results, circularity not so much influences the health effects of the hazards, but rather their presence depending on the persistence and transfer of food safety hazards in a circular system.

## Introduction

Over the last century, food production within Europe focused on increasing yields so as to feed as many people as possible. As a result, agricultural production has become very effective and Europe moved from a net agri-food importing to an exporting continent. However, the downside of this development is the adverse impact of agriculture and food production on the environment due to the increased greenhouse gas emissions as well as biodiversity loss and water pollution^1^. To counteract this impact, the European Commission (EC) has deployed a new growth strategy that reconciles the economy with the planet, which is implemented in the EC Green Deal. Connected to the Green Deal is the EC Farm to Fork strategy focusing on a transition towards shorter supply chains, minimising pesticide use, reducing food loss and waste, and increasing circular production (e.g. re-use of side streams)^2^. This implies that food waste should be prevented as much as possible and, if not possible, be re-used within the food system thereby closing the production loop^1,3^. Globally, between 11% and 60% of the food is wasted somewhere along the food supply chain^4^. From farm-to-fork, waste is generated both in plant and animal production systems^3^. Food production can lead to by-products such as crop residues, side streams obtained during food processing, food waste as well as human and animal faeces. These by-products still contain valuable nutrients that can be applied in food production. For example, crop residues and faeces can be used as fertilisers in agriculture^5^. Furthermore, food waste and side streams from food production could be reused as animal feed in livestock production^6^.

Apart from the expected positive environmental and socio-economic impacts, a circular economy can also have consequences for food safety. By-products that have not been reused in food production till today do not have a history of safe use. Possible safety issues are largely unknown and, therefore, need to be assessed prior to reusing by-products within the production process^7,8^. Known hazards may occur at unexpected steps in the production system, or relative new hazards may be introduced. Furthermore, when closing the loop, chemical hazards that are present at low levels in the biomass may accumulate when reusing. This could lead to elevated levels of chemical hazards in final foods that may impact human health^9^. The key challenge in the transition towards a circular economy is, thus, to continue meeting the current food safety standards while closing the production loops.

It is important to think upfront about possible food safety problems that may be encountered with the transition from linear production chains towards circular production cycles. Therefore, the aim of this research was to develop a framework that can be used for a priori assessment of food safety in the transition towards circular food production systems in Europe. The framework is based on the principles of Hazard Analysis Critical Control points (HACCP). Following the General Food Law (EC/2002/178), food business operators (FBO) need to have a HACCP system in place. Here, the framework focuses on the full supply chain, rather than on one FBO. The study focuses on European situation and area, driven by recent adoption in European policy, such as the Grean Deal and the Farm-to-Fork strategy. Results from applying this framework could be used to identify appropriate actions to prevent from human health risks.

## Results

### Case study 1: use of animal manure in agriculture

#### Step 1. Describing the changes in the food supply chain as a result of the circularity transition

Artificial fertilisers are fossil-based and are being applied to improve the quality of the soil. The transition from a fossil-based economy towards a more circular production system applying renewable sources requires the reuse of waste streams. Replacing artificial fertilisers with animal manure contributes to the goals of a circular economy^5^. Animal manure has been applied for cultivating cereals and feed crops, and currently, broader applications, for example in horticulture, are being explored^10^. Although in the future other waste streams, such as sewage sludge, may be applied in European crop cultivation, this case study focused on replacing artificial fertilisers with animal manure in horticulture. Other sources that may contaminate crops, such as irrigation water, were not included in this case study. Furthermore, the focus of the study was on human health so other effects, such as ecotoxicity, were not considered.

#### Step 2. Identifying potential food safety hazards

For the treatment of diseases in livestock a range of pharmaceuticals are being applied such as antibiotics, antiparasitics, coccidiostats and hormones. These are subsequently excreted in the faeces of the production animals^11,12^. Recent studies showed that these substances may persistently be present in animal manure and can subsequently be present, and in some cases persistent, in the soil^11–14^. When the manure is applied on arable land, these chemicals have the potential to be taken up by the crops^15^. Also, they contribute to the selection of resistant bacteria in the soil, which can be transferred to edible parts of the crop as well^16^.

Various foodborne pathogens may also be excreted in livestock manure: *Campylobacter* spp., *Clostridium* spp., *Listeria* spp., *Escherichia coli* and *Salmonella* spp.^12^. Studies have shown that these pathogens may survive for a considerable amount of time in manure and can, once applied on arable land, subsequently be transmitted to the crops grown on this land^17,18^.

#### Step 3. Prioritising food safety hazards related to the circularity transition

In step 3 of the framework, the food safety risks related to a circular economy are evaluated based on occurrence and severity of the hazards. This case study focused on the food safety hazards for which data were available to establish the occurrence in manure, the persistence in manure and the soil, and the subsequent transfer to crops. Therefore, the antibiotic groups tetracyclines, sulphonamides, (fluoro)quinolones and macrolides, and the pathogens *Salmonella* spp. and *E. coli* in raw manure were considered. Data used for the classification were limited in some cases (e.g. only two substances within the group of macrolides were examined) and not always the same substances within the groups of antibiotics were studied. The classification should thus be seen as exemplary to how a full assessment could be done.

##### Occurrence

An extensive review on the prevalence of various microbiological and chemical hazards in manure showed a wide variability in the occurrence of the hazards and also between the different manure types. It should be noted that the below-average concentrations were based on a global review^12^. For the antibiotic groups included in this study, highest concentrations in raw manure were found for tetracyclines (average of 53,537 ng/g dm) and (fluoro)quinolones (average of 37,566 ng/g dm), whereas lowest levels were found for sulphonamides (average of 6048 ng/g dm) and macrolides (average of 3601 ng/g dm). In Europe, (fluoro)quinolones, apart from flumequine, are seen as third choice antibiotics and are thus used at relatively low frequency in animal production resulting in – in general - lower concentrations in manure^19^. Average concentrations of pathogens in raw manure from cattle, swine or poultry range between 4E4 and 1E8 cfu/g dm for *E.coli*, and between 4E3 and 3E5 cfu/g dm for *Salmonella* spp. depending on the origin of the manure^12^. Results from this review are summarised in Table 1.

**Table 1:** Occurrence of antibiotics and pathogens in manure^a^

Hazard	Average concentration		
	Raw cattle manure	Raw poultry manure	Raw swine manure
Chemical hazards (ng/g dm)			
(Fluoro)quinolones	7900	92696	12099
Macrolides	40	3836	6927
Sulphonamides	1277	10591	6277
Tetracyclines	40805	30166	89639
Microbiological hazards (cfu/g dm)			
* E. coli*	1.3E8	2.2E7	4.7E4
* Salmonella*	3.1E5	1.4E4	3.7E3

^a^Based on Ghirardini et al.^12^.

##### Persistence

Based on the paper from Lahr, Bondt^14^, the persistence of antibiotic residues both in manure and in the soil, expressed as DT50 (time needed to reduce the compound by 50%), was classified as: Low: <30 days, Medium: 30–100 days and High: >100 days. The average DT50 of groups of antibiotics (tetracyclines, sulphonamides, fluoroquinolones and macrolides) in both manure and soil were used^11,13^ to prioritise the antibiotics (see Table 2). Sulphonamides relatively quickly dissipate in all manure types, with an average DT50 between 1 and 12 days. On the other hand, (fluoro)quinolones are much more persistent (DT50 75- > 365 days), but half-life depends on the manure type^11^. Persistence in the soil shows a similar picture: quinolones are far more persistent than sulphonamides^13^. Therefore, in general (depending on manure and soil type), quinolones are classified in the ‘High’ group, followed by tetracyclines, macrolides and sulphonamides (see Table 3).

**Table 2:** A. Average DT50 values for groups of antibiotics in various manure types (low (score 1): <30 days, medium (score 2): 30–100 days, and high (score 3): >100 days)^a^; B. Average DT50 values for groups of antibiotics in two soil types (low (score 1): <30 days, medium (score 2): 30–100 days, and high (score 3): >100 days)^a^

A	Cattle solid	Cattle semi-solid	Cattle liquid	Pig	Broiler
(Fluoro)quinolones	564	226	82	75	121
Macrolides	29	47	9	50	61
Sulphonamides	1	3	12	2	3
Tetracyclines	76	57	32	14	33

B	Sand	Clay
(Fluoro)quinolones	182	97
Macrolides	56	73
Sulphonamides	1	1
Tetracyclines	9	1

^a^As based on Berendsen et al.^11^.

**Table 3:** Prioritisation of food safety hazards when reusing manure in horticulture

Hazard	Occurrence in manure	Persistence manure	Persistence Soil	Transfer to crops^a^	Likelihood of presence in crops	Severity	Prioritisation
*Chemical hazards*						*Based on ADI and antibiotic resistance*^b^	
(fluoro) quinolones	High	High	High	Low	High	High	High
Macrolides	Low	Medium	Medium	Low	Low	Medium	Low
Sulphonamides	Low	Low	Low	High	Low	Low	Low
Tetracyclines	High	Medium	Low	Low	Medium	Medium	Medium
*Microbiological hazards*						*Based on DALY*^c^	
*E. coli*	High		Low	Medium	Medium	Medium^d^	Medium
*Salmonella*	Low		Low	Medium	Low	Medium	Low

^a^Weighed to a lesser extent in the likelihood of presence than the occurrence and persistence in manure and manure-amended soil.

^b^Van Asselt et al.^24^.

^c^Bouwknegt et al.^57^, see Table 4.

^d^Based on STEC.

Persistence of the pathogens *E.coli* and *Salmonella* spp. in manure-amended soil has been reviewed by ref. ^20^. The study showed that persistence is temperature dependent with higher persistence at lower temperatures. At medium temperature conditions, the time for 1 log reduction in the field is on average 12.6 days for *E.coli* and 15.9 days for *Salmonella* spp.^20^. This shows that persistence of these two pathogens is comparable. GLOBAL GAP indicates a waiting time of 60 days between applying raw manure and harvest of leafy greens^21^. This implies a 4–5 log reduction of *Salmonella* spp. and *E.coli* can be achieved. As a result, persistence was classified as low.

##### Transfer

Transfer of chemical hazards from manure to the plant depends on many factors, which are not yet completely understood. For simplicity reasons, in this study, we assume that antibiotic uptake can be expressed by the *K*_OC_, the organic carbon-water partition coefficient. A *K*_OC_ value below 1 implies the substance is water soluble and thereby can readily be taken up by the plants. Pot experiments showed that only the group of sulphonamides is likely to be readily taken up by plants with a log *K*_OC_ value of around 0.5^13^. Transfer for this group of substances is thus classified as a high likelihood of transfer, whereas the tetracyclines (log *K*_OC_ ≥ 3.0), (fluoro)quinolones (log *K*_OC_ ≥ 2.9) and macrolides (log *K*_OC_ ≥ 2.0)^13^ were classified as low likelihood of transfer.

Soil and animal manure are important sources of pre-harvest crop contamination^22^. Nevertheless, although *E.coli* can survive in the soil over a long period of time and can be taken up by the plants, the transfer from manure-amended soil to the edible parts of the plants is limited as is recently demonstrated for leafy vegetables. However, pathogens may still contaminate crops through soil splashing during heavy rainstorms^23^. As such, transfer to crops is classified as medium. Since the survival and persistence of *Salmonella* spp. and *E.coli* are comparable and both are Gram-negative bacteria, it is expected that their transfer behaviour is also comparable.

##### Health effect

Apart from the presence, the health effects of the hazards need to be evaluated as well. For this purpose, toxicity factors identified in an earlier study were used^24^. Both chronic toxicity to humans (ADI) and antibiotics resistance were incorporated. In case an antibiotic was classified as second or third choice antibiotic^24^, the substance was classified as having a high health effect. If both chronic toxicity and antibiotics resistance were classified as high effect on human health, the severity was classified as high. If either chronic toxicity or antibiotics resistance were classified as high, the overall classification for severity was seen as medium effect on human health, and when both were classified as low, the severity was classified as low. For the microbiological hazards, the DALY concept was used as indicated in Table 4.

**Table 4:** Severity score for pathogens based on DALY/case^a^

Pathogen	DALY/case	Classification
*Bacillus cereus*	0.0023	Low
*Clostridium perfringens*	0.0032	Low
*Cryptosporidium* spp	0.0024	Low
*Giardia* spp	0.0018	Low
*norovirus*	0.0026	Low
*Rotavirus*	0.0054	Low
*Staphylococcus aureus*	0.0026	Low
*Campylobacter*	0.034	Medium
*Hepatitus A virus*	0.161	High
*Hepatitis E virus*	0.434	High
*STEC O157*	0.065	Medium
*Salmonella* spp	0.039	Medium
*Listeria monocytogenes*	1.161	High
*Toxoplasma gondii*	3.173	High

^a^Based on Bouwknegt et al.^57^.

##### Prioritisation

Table 3 summarises the results of the classification of hazards based on their occurrence and severity. The likelihood of finding food safety hazards in crops when reusing animal manure in horticulture was assessed by evaluating the presence of these hazards in manure, their persistence in both manure and in manure-amended soil, and their potential transfer to the crop. The use of *K*_OC_ values for transfer is a simplification of the reality and as such this evaluation was weighted less in the overall classification. Overall, (fluoro)quinolones were estimated as having the highest likelihood of presence in crops, followed by a medium classification for tetracyclines and *E.coli*. Macrolides, sulphonamides and Salmonella were evaluated as low likelihood of presence based on low occurrences ad persistence in manure and manure-amended soil. Since (fluoro)quinolones were also classified as high severity, they were prioritised as highest risk in this case study. Note that severity in this study only reflects the effects on human health due to food consumption and not on the environment.

### Case study 2: using side streams for insect rearing for feed and food

#### Step 1. Describing the changes in the food supply chain as a result of the circularity transition

In the last three decades, insects are considered an alternative protein source for feed and food production in Europe. One of the most investigated and promising insect species to date is the black soldier fly (BSF, *Hermetia illucens* L.). BSF has a high potential to be grown at the commercial (mass) scale as an ingredient for feed and food production. BSF can feed efficiently on a wide range of waste and side streams, has high nutritional value, and low emission of greenhouse gases and ammonia^25^. In Europe, insects are considered farm animals and, therefore, they fall within the scope of the ‘feed ban’ (Regulation (EC) No 999/2001), which prohibits the use of farmed animal-derived proteins in feed for ruminant and monogastric animals. Since the 1^st^ of July 2017, the use of insect proteins from seven insect species in feed for aquaculture animals has been authorised (Regulation (EU) No 2017/893), amongst which BSF. Later on, following Regulation (EC) (EU) 2021/1372, the use of insects as feed for poultry and pigs is also allowed. Regarding feed for insects, according to Regulation (EC) No 999/2001, BSF and other insects may only be reared on feed materials and compound feeds that are also allowed to be used as feed for other production animal species. These include vegetable feed materials (class A) and (former) foods produced for human consumption, without meat and fish (class B1). So, given the alternative use of the current substrates used to feed insects, insect rearing can currently not be considered sustainable^25^. Should BSF be grown on a mass scale to contribute to a circular economy in an economic and sustainable way, it should be possible (legally allowed) to rear them on waste and side streams which have little or no alternative use for feed and food production^26^. It is expected that in the near future, some of such other substrates will also be allowed to be used for insect rearing, starting with (former) foods produced for human consumption, with or without meat and fish (class B2), followed by by-products from slaughterhouses (hides, hair, feathers, bones etc.) that do not enter the food chain but originate from animals fit for human consumption (class C)^25,27^. The use of food waste from food for human consumption of both animal and non-animal origin from restaurants, catering and households (class D) and animal manure and intestinal contents class (E) are not expected to be allowed for the rearing of insects in the near future in Europe, while the use of other types of organic waste of vegetable nature such as gardening and forest material (class F) and human manure and sewage sludge (class G) is even more far away^27^.

#### Step 2. Identifying potential food safety hazards

During insect rearing, food safety hazards can accumulate from the substrates into the insects; these can be microbiological and chemical hazards present in the substrate, and – when feeding upon them – the insects consume or are contaminated with the food safety hazards as well. Depending on the excretion and metabolic pathways by the particular insect species, food safety hazards can either accumulate in the insect body, or can be downgraded and/or excreted. In case of accumulation, the hazards can be present in the insect larvae at harvest, or can be present on their outsides. From recent research it is, for example, known that cadmium accumulates in BSF larvae, and arsenic accumulates in Lesser meal worms (Alphitobius diaperinus), but not the other way around^28^. In a recent review, it is reported that the accumulation of chemicals in insects depends on a wide range of factors, amongst which, the insect species and its life stage at harvest, the particular hazard, and the substrate on which the insects are reared as well as the particular rearing conditions^29^.

#### Step 3. Prioritising food safety hazards related to the circularity transition

In order to prioritise food safety hazards related to a change in substrate used for rearing insects, the presence of the hazard in the substrate as well as possible accumulation/excretion of the hazard by the insect species, given the rearing conditions, should be established. Thus, the presence of the hazard in the harvested insects can be estimated by the presence in substrate x transfer to and persistence in the insect. In this case study we focus on BSF as it is currently the most interesting species for rearing on alternative substrates (not allowed yet) in the near future in Europe since this species can thrive on a large variety of substrates. In particular, we focus on the use of household waste and animal manure, instead of the currently allowed substrates, to feed BSF reared commercially for feed/food in Europe. Furthermore, given the lack of data on the fate of microbiological hazards in BSF, we focus on chemical contaminants, in particular the groups of dioxins and PCBs, mycotoxins, pesticides (as a group), and heavy metals.

##### Occurrence

Various chemical hazards may be found in the possible substrates of household waste and manure used for the rearing of BSF. Dioxins and PCBs may be found in low amounts in household food waste (140 + /- 20 pg/kg WHO-TEQ (unpublished data WFSR). Dioxin-like compounds were also found in pig manure (40–62.4 µg eq TCDD/L)^30^, therefore, occurrence in household waste and in manure was considered Low and Medium, respectively (Table 5). Veterinary drug residues may be found in animal manure at various levels, depending on the origin of the manure. Case study 1 also showed that for antibiotics, the levels in manure vary depending on the antibiotics group. High levels may be found for tetracyclines, therefore, as a worst case, the occurrence of veterinary drug residues in manure was considered High. In household waste, veterinary drug residues are not likely to be present, so occurrence was considered Low (Table 5). Recent research showed that pesticides may be found in household waste, but levels are low (between 0.011 and 0.05 mg/kg, unpublished data WFSR). Pesticides are not expected to occur at relevant levels in manure, hence, occurrence in manure was considered Low (Table 5). The presence of heavy metals was found to be low in manure. In household waste, higher levels were found (unpublished data WFSR), with a concentration of Cadmium at 0.127 mg/kg and Lead at 43.5 mg/kg (ML is 1 or 2 mg/kg for Cadmium in animal feed of vegetable or animal origin, respectively, and 10 mg/kg Lead in feed (Directive 2002/32/EC).

**Table 5:** Prioritisation of chemical food safety hazards when rearing black soldier fly larvae on side streams

Chemical hazard	Occurrence in household waste	Occurrence in manure	Accumulation in insects	Severity	Prioritization
Dioxins & PCBs	Low	Medium	Medium	High	Medium
Tetracyclines/veterinary drugs	Low	High	Low	Low	Low
Pesticides	Low	Low	Low	Low	Low
Cadmium	Medium	Low	High	High	High
Lead	High	low	Medium	High	High
Aflatoxin B1	Low	Low	Low	High	Low
Deoxynivalenol	Low	Low	Low	High	Low

The main mycotoxins in terms of presence in commodities grown in Europe are considered aflatoxin B1 and deoxynivalenol (DON). For food products, legal limits have been set in Europe for the maximum presence of these two mycotoxins (and some others) in food products. For aflatoxins, legal limits also have been set for their presence in feed materials, whereas for DON, guidance values have been set for the presence of this toxin in feed materials. The presence of DON and aflatoxin B1 in household waste (food waste from food for human consumption of both animal and non-animal origin from restaurants, catering and households) is expected to be low, since all food needs to comply with the legal limits for the presence of mycotoxins. Also, in animal manure, these mycotoxins are expected to be present in low or negligible amounts.

##### Persistence

Depending on the waiting time before using household waste or animal manure as substrate for BSF rearing, some of the considered chemicals may already be partly degraded in the respective materials. This effect is not further considered in this case study, rather we focus on the possible transfer and accumulation of the chemical in the BSF when reared on the considered side stream.

##### Transfer

Meyer et al. (2021)^29^ reviewed the accumulation of chemical hazards in insects, using bio-accumulation factors. Their review showed that heavy metals may accumulate from the substrate in insects. Cadmium showed to accumulate in the BSF larvae; in some instances a high bio-accumulation – up to factor 20^28,29^- was found. The bio-accumulation factor of Lead was found to be up to 2.3^29^. Dioxins and dl-PCBs were found to accumulate to little extent, with a BAF up to a factor of two^28,29^. Veterinary drug residues seem not to accumulate in BSF, although low residue levels of some veterinary drugs have been found in BSF. Also, some veterinary drugs are readily degraded by BSF. For instance, Cai, Ma^31^ showed that BSF could rapidly degrade tetracyclines (spiked to wheat bran). Investigated mycotoxins, including aflatoxin B1, deoxynivalenol, Zearalenone, Ochratoxin A, and T-2 and HT-2 toxins, do not accumulate in BSF larvae^29^. Moreover, aflatoxins seem to be downgraded by BSF larvae. Pesticides are also shown not to accumulate in BSF^29,32^.

##### Health effect

For scoring the health effects of the chemical hazards, health-based guidance values are used. Dioxins were thus classified as high severity (Tolerable Weekly Intake (TWI) is 2 pg/kg bw^33^) as well as Cadmium (TWI is 2.5 µg/kg bw^34^) and lead (BMDL_10_ is 0.63 µg/kg bw/day^35^). Pesticides in general have ADIs or Acute Reference Dose (ARfD) that are above 10 µg/kg/day (EU Pesticides Database), which can be classified as low. Tetracyclines have a low toxicity according to JECFA^36^. Aflatoxin B1 has a BMDL_10_ of 0.4 µg/kg bw/day^37^, which is classified as high severity. Deoxynivalenol is also classified as high severity since the group TDI is 1 µg/kg bw^38^.

##### Prioritization

The prioritization of chemical food safety hazards in BSF larvae was established based on the occurrence of the contaminants in the considered waste stream, the transfer and accumulation (persistence) of the chemical in the insect larvae, and the toxicity of the particular chemical to human health. Combining these factors ultimately leads to a high priority for the heavy metals Cadmium and Lead, a medium priority for dioxins and PCBs, and a low priority for the mycotoxins aflatoxin B1 and deoxynivalenol, for tetracyclines and veterinary drug residues in general, and for pesticides (Table 5).

### Case study 3: local production/shorter supply chains

#### Step 1. Describing the changes in the food supply chain as a result of the circularity transition

Consumer demand for ‘local’ products has led to the development of a diverse range of local food networks and short food supply chains (SFSCs), such as, farmers’ markets, ‘farm-gate’ sales, and basket/box delivery systems. Each individual local food initiative has evolved in the context of the place in which it operates, the food products it markets, and the nature and location of its consumer base^39^. As an example country, in the Netherlands, the number of agricultural farms that sell products via a short chain (direct to consumer or with only one chain stage in between) is increasing fast. In 2017, in total 10.5% of the agricultural companies in the Netherlands sold products via the short chain, the majority of the farms selling products directly to the consumer. This percentage increased to 13.7% in 2020^40^. The percentage of short chains in organic agriculture (39%) is larger than at conventional production systems (13%). Also, with organic agriculture, products are sold more locally as compared to conventional products^40^. In addition to organic production, circular production often occurs or is considered at the local or regional level^1^.

#### Step 2. Identifying potential food safety hazards

In terms of food safety, short supply chain actors should comply to the same legal requirements as conventional (long) supply chains actors. However, additional private system requirements often do not apply to the short supply chain. In local supply chains, the number of persons involved with quality assurance (including food safety) is lower than in conventional chains, and their knowledge on quality systems or food safety may be lower as well. On the other hand, the use of quality management systems, like the Hazard Analysis Critical Control Point (HACCP) system, which is necessary for large production plants involving many people in long supply chains, may be less needed for farmers producing at the local scale. The use of good practices, as foreseen in EU food hygiene legislation, might be sufficient in this case.

Food products that are produced either locally or via the short chain do not necessarily have a lower probability of contamination. With local production of feed and food, ingredients are sourced from the region. Thus, in case of local contamination, in a particular area, the probability that the produced foods are contaminated is higher. This is because with the mixing of ingredients from different origins, contamination of one of the ingredients (in case present) will be diluted. As an example, locally produced wheat flour, from local wheat farmers, sold to the consumer may contain more mycotoxins than wheat flour produced from mixing wheat from different origins. This is because the fungal infection and mycotoxin production in grains may be a very local problem, mostly depending on local weather conditions. This implies that if batches from agricultural products are contaminated, they will end up in the products sold at the farm or local market. This may be the case for pathogenic contamination, when conditions for growth of the pathogen locally occur, and for chemical hazards which can have a local source, such as dioxins, heavy metals and mycotoxins.

#### Step 3. Prioritising food safety hazards related to the local food production

As a case study, we focus on mycotoxin contamination in locally produced wheat in the Netherlands, and prioritised the following regulated mycotoxins in Dutch wheat flour: Deoxynivalenol (DON), T-2 and HT-2 toxin, and Zearalenone (ZEA). Two scenarios were compared: (1) a conventional scenario where batches of wheat flour originated from 5 to 10 different fields, and (2) a short-chain scenario in which a batch of wheat flour originated from one local field only. Factor 1 (occurrence of hazards) is the main factor of importance for this case study. Factors 2 (the persistence) and (3 (the transfer) are less relevant for this case study since both the persistence of the mycotoxins in the product and the transfer of the mycotoxins from the wheat kernels to the wheat flour are expected to be similar in case of wheat made from locally grown wheat or from a mixed batch of wheat.

##### Occurrence

Information on levels of the mycotoxins under study were based on samples that have been collected from commercial wheat fields at harvest in the Netherlands between 2009–2018. The description of this dataset can be found in Van der Fels-Klerx, Focker^41^. A summary of the data is presented in Table 6. Mycotoxin levels shown in Table 6 are levels measured in the unprocessed wheat kernels at harvest in different fields in the Netherlands in the study period. We considered local wheat flour to come from one local field, whereas conventional wheat flour to come from at least 5 different fields, up to 10 different fields, all situated in the Netherlands. For the short-chain scenario, one concentration was drawn from the data summarised in Table 6. This was repeated 100,000 times. For the conventional scenario, first a number n of fields that are mixed together was determined, with n being a random number between 5 and 10. Then n concentrations were drawn from the data summarised in Table 6, which were then averaged. This was repeated 100,000 times. The results are shown in Table 7. The average estimated mycotoxin concentrations in wheat kernels were similar for both scenarios. The median and the percentage of batches exceeding the EC maximum limit (ML) for DON and ZEA or the guidance level of T-2 and HT-2 toxins (for the respective mycotoxins) were higher for the conventional scenario as compared to the short-chain scenario. However, the maximum concentration was higher for the short-chain scenario compared to the conventional scenario. A threshold of 3% of the cases having a concentration above the EC limits was applied to classify the occurrence into the classes High or Low^42^.

**Table 6:** Concentration of mycotoxins in Dutch wheat in the period 2009–2018^a^

	Mycotoxin concentration (µg/kg)		
	DON	T-2/HT-2	ZEA
Median	60	10	25
Mean	411	11.7	65
Max	15400	66	2000
% >ML^b^	6.1	0.0	6.9

^a^Based on results of 293 commercial wheat fields in the Netherlands (Van der Fels-Klerx et al.)^41^.

^b^Maximum Limit (ML) for respectively DON and ZEA in unprocessed wheat being, respectively, 1250 µg/kg and 100 µg/kg (Regulation (EC) No 1881/2006), and recommended level for T-2 and HT-2 in unprocessed wheat, being 100 µg/kg (Recommendation 2013/165/EU).

**Table 7:** Mycotoxin concentration (µg/kg) in the raw wheat product (wheat kernels at harvest)

	Min	Median	Mean	Max	% >ML^a^
Short-chain scenario					
DON	25	60	411	15,400	6.2
ZEA	25	25	63	2000	6.7
T-2 + HT-2	10	10	12	38	0.0
Conventional scenario					
DON	45	221	409	5257	7.5
ZEA	25	33	65	1001	13.7
T-2 + HT-2	10	11	12	66	0.0

^a^Maximum limit (ML) for respectively DON and ZEA in unprocessed wheat being, respectively, 1250 µg/kg and 100 µg/kg (Regulation (EC) No 1881/2006), and recommended level for T-2 and HT-2 in unprocessed wheat, being 100 µg/kg (Recommendation 2013/165/EU).

##### Persistence

Mycotoxins are hardly degraded during processing of raw materials into final products, therefore, the persistence is considered high.

##### Transfer

By milling wheat kernels to make wheat flour, the concentrations of mycotoxins are lowered. However, mycotoxins are not removed. Processing factors were seen as a measure for transfer of mycotoxins from the wheat kernels to the flour. Schaarschmidt & Fauhl-Hassel^43^ concluded on a processing factor for DON of between 0.5 and 0.8 for white flour. The processing factors were, in general, higher for wholemeal flour (between 0.7 and 0.9). For ZEA, the processing factor for white flour was between 0.1 and 0.9, whereas it is between 0.2 and 0.4 for T-2 and HT-2 toxins. Since a significant percentage of mycotoxins were transferred to the final product, the transfer of mycotoxins from the wheat kernels to the wheat flour was classified as high. As indicated previously, persistence and transfer were assumed to be the same for the conventional and the short-chain scenario.

##### Health effects

The severity of effects of mycotoxins to human health was based on the TDI, with the following TDIs for the mycotoxins of interest: a group TDI of 1 µg/kg bw/day for DON^38^, a group TDI of 0.25 µg/kg bw/day for ZEA^44^ and a group TDI of 0.02 µg/kg bw/day for T2 and HT-2^45^. Since these TDI’s were all below 10 µg/kg bw/day, these hazards were all classified as high risk.

##### Prioritization

Since the only difference related to mycotoxins in the two scenarios for the wheat chain (short supply chain vs conventional chain) is their occurrence, the prioritization is primarily based on this factor. Overall, DON and ZEA are classified as high priority, and T-2 and HT-2 as medium priority (Table 8).

**Table 8:** Prioritization of mycotoxins in wheat flour in the short-chain scenario

Mycotoxin	Occurrence in the raw product	Persistence	Transfer from raw product to finished product	Severity	Prioritization
DON	High	High	High	High	High
ZEA	High	High	High	High	High
T-2 and HT-2	Low	High	High	High	High

## Discussion

Circular food systems are more complex than linear food production chains and the transition from linear to circular food system may lead to the introduction of new food safety hazards or the accumulation of known hazards into the final product. Therefore, prior to closing production loops, it is essential to identify potential hazards, prioritise these and take appropriate actions to mitigate or control them during food system change^7^. For this purpose, a framework was established in this study that can help in the initial (design) phase of implementing a circular food system. Within the framework, food safety hazards are prioritised based on their potential human health risk, based on the presence of the hazard in foods and effects and human health. These two factors, and their underlying indicators, were classified in low, medium and high, using predefined thresholds. Thresholds were needed to determine whether a factor or an indicator is classified into either low, medium, or high, as based on the available information or values on the respective factor/indicator. The disadvantage of this method is that the establishment of such thresholds is subjective and may influence the outcome of the classification. However, the data used to obtain the classification are objective and the method applied is transparent. Furthermore, the approach followed is easy to apply and communicate^46^. Given the low availability of data, often associated with new systems, this method was considered appropriate. When more data will become available in future, the current case studies can be populated with more data, or a more quantitative approach could be taken.

Three case studies were performed to explore the usefulness of the developed framework. Since the case studies were meant for demonstrative purposes, a limited number of food safety hazards were selected for the prioritization task. Primarily those hazards were selected for which enough data were available for the prioritization. However, since the framework uses classes ranging from low, medium to high, it also allows for the inclusion of hazards with limited data. In that case, expert elicitation is needed to perform the prioritization. The case studies performed showed that circularity in the food systems does not influence the severity of the hazards, but rather their occurrence and thus also their final priority. The case study on locally produced wheat flour showed that the average occurrence was not relevant, but rather the incidental high concentrations found (Table 6). Since presence of the hazard is the predominant factor, indicators for food safety should focus on changes in the presence of food safety hazards when moving towards a circular food systems, or comparing a linear to a circular system. Presence in the final food products depends on several sub-factors, expressed as measurable indicators, such as occurrence in the raw material, persistence in the waste stream and/or the environment and subsequent transfer to the edible parts of the plant or animal. The case studies on reusing waste streams (i.e. using animal manure for horticulture and using waste materials as substrate for insect growth) indicated that estimating the values of these factors may need assumptions. For the manure case, transfer was estimated based on the *K*_OC_-value as a measure for mobility of the compound. However, both transfer and persistence depend on the soil composition, the crop characteristics and the physico-chemical properties of the chemical^47^. Some crops, thus, take up chemicals more easily than others^48–50^. A more detailed estimation of the effect of manure use in crop production is, therefore, needed to obtain a more accurate prediction of microbial and chemical hazards to be expected in the final product. However, the case study applied here was for demonstration purposes only – to test the feasibility of the designed framework - and aimed for a rough indication of a low, medium or high risk related to changed agricultural practices. As such, the use of *K*_OC_ and DT50 values are appropriate to prioritise hazards that need further evaluation and assessment.

The case study on local food production showed that although there are many advantages when producing and selling food locally, local contamination of crops potentially leads to contaminated food products that would otherwise have been diluted when mixed with crops from other regions. As is the case with circularity, local production also does not so much affect the severity of the hazards, but rather the presence of the hazard and, thus, the final priority of the hazards.

The case studies also demonstrated that it is possible to identify and classify hazards within a case study, but it is more difficult to compare hazards between case studies as not always all indicators are taken into account. The case study focusing on local production, for example, needed a different approach than the cases on manure and insects. Nevertheless, the case studies showed that the framework is useful in identifying and prioritising food safety hazards in circular food producing systems and can be applied in a broader perspective.

In conclusion, in this study, a framework has been developed for identifying and prioritising potential food safety hazards that may occur when changing from linear to circular food systems. This framework may be applied at the design phase of reusing waste materials and side streams in order to prevent potential food safety issues during the transition towards circular food production systems. Since the elements to include are case dependent, the framework should be applied on a case-by-case situation. The framework has been tested in three case studies showing that circularity not so much influences the health effects of the hazards, but rather their presence in foods, depending on persistence and transfer of food safety hazards in a circular system.

## Methods

### Steps of the framework

**Fig. 1:**
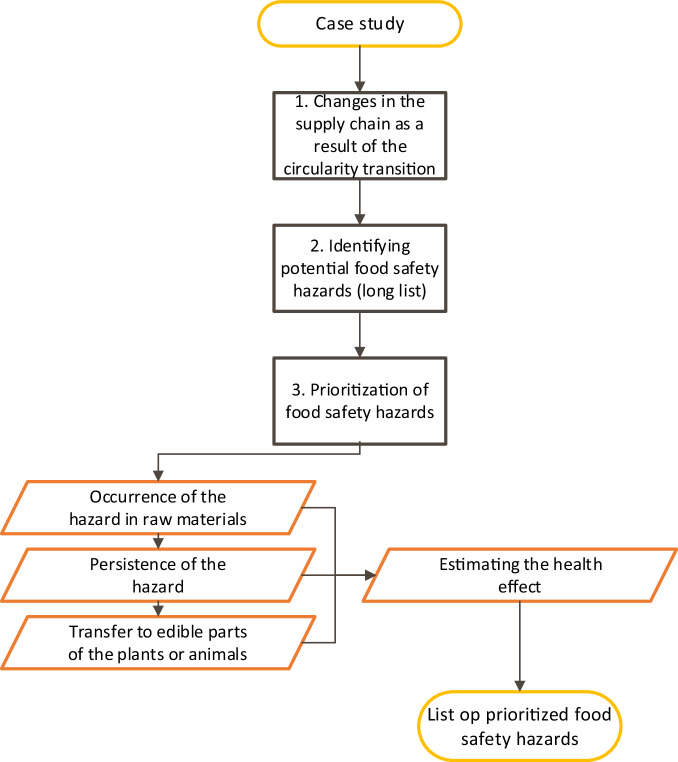
Framework to prioritise food safety hazards related to circular food systems.

In order to identify and prioritise possible food safety hazards in a circular food production system, a framework consisting of the following three steps has been developed (see Fig. 1):

1. Describing the changes in the food supply chain as a result of the circularity transition

In order to identify potential hazards, it is relevant to first describe the changes in the supply chain related to the transition towards the circular system and compare these with the baseline (the current linear production chain).

2. Identifying potential food safety hazards

Once the transition is described, the possible food safety hazards (known and new hazards) that may emerge from the new situation need to be identified.

3. Prioritising food safety hazards related to the circularity transition

Once a list of potential chemical and microbiological food safety hazards has been identified in step 2, the hazards need to be ranked based on their potential human health risks.

### Changes in the food supply chain as a result of the circularity transition

First, the baseline situation needs to be described, which is the current supply chain under study, with its stages, processes within stages, and flows of materials between stages. Given this baseline situation, the proposed changes to close production loops needs to be added, such as to make the current supply chain circular. Also, it needs to be identified how the proposed changes affect the stages in the production chain, processes and production flows. This step needs knowledge from the supply chain under study, with expert information from stakeholders of the particular chain.

### Identifying potential food safety hazards (long list)

The closure of production loops may result in the emergence of food safety hazards, which are introduced with closing the production loop to arrive at a circular supply chain. First, it may result in the possible accumulation or (increased) presence of known hazards. Second, it may result in the presence of relatively new, not yet fully known, hazards introduced with the commodity now used in the circular supply chain.

To identify potential food safety hazards in the circular supply chain, systematic reviews can be used, to gather all up to date knowledge and/or expert elicitation studies. The decision to use either of them, or their combination, depends on the data available from literature in combination with available resources and time for the study. For systematic reviews, standardised protocols are available such as from EFSA (2010). For elicitation of expert judgments, a wide range of methods are available, ranging from in-depth individual expert interviews, to group workshops and Delphi approaches^46,51^. The choice of the type of method to use depends on the particular question at hand, how much information is already available, whether or not consensus on the list of hazards is needed etc.

The identification of possible food safety hazards in the circular supply chain results into a long list of possible hazards. These then need to be prioritised in the next step to identify the most important food safety hazards.

### Prioritization of food safety hazards

#### Food safety risks

According to the Codex Alimentarius (2004), a food safety risk is defined as a function of the probability of an adverse health effect and the severity of that effect, consequential to the occurrence of the hazard in food. A food safety hazard is a biological, chemical or physical agent (or condition of) food with the potential to cause an adverse human health effect. Food safety risks are thus related to: the occurrence of food safety hazards in foods, the human intake of the foods, the probability of health effects, and the severity of those effects.

Indicators are available to identify and prioritise food safety hazards, based on their human health risks through food consumption. A literature review revealed that various methods are available for risk ranking of food safety hazards based on their impacts on human health^46,51^. The various methods all consider both the *presence of the hazards* in food and the *health effects related to* this presence of the hazard in foods, but the methods vary in the parameters or indicators they take into account. Methods furthermore range from quantitative to qualitative approaches.

In this study, a semi-quantitative approach is used since we expect a lack of data due to the limited studies performed so far on the effects of circularity on food safety. The following sections describe the estimation of the presence and health effects of food safety hazards in order to estimate the human health risks related to consumption of foods produced in circular systems.

#### Estimating the presence of food safety hazards

**Fig. 2:**
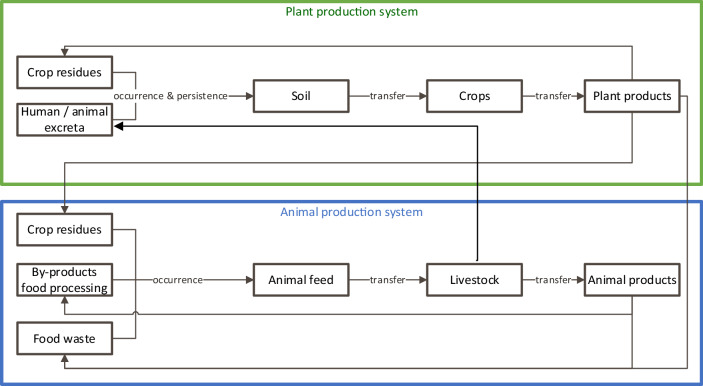
Hazard routes in circular food production.

The various routes through which the presence of food safety hazards in foods may be influenced in a circular food production system are depicted in Fig. 2. Indicators that are relevant to estimate the presence of food safety hazards in the final food product as a result of using by-products or closing production loop are:

1. Occurrence of the hazards in raw materials

The occurrence of food safety hazards in the raw materials, i.e. the materials used as starting point in circular food production is of relevance to estimate the potential accumulation (or dilution) throughout the circular agriculture and food production system. These may entail, for instance, crop residues, by-products from food processing (including packaging materials), human/animal faeces or food waste.

2. Persistence of the hazard

The persistence of a hazard reflects the level of degradation of a hazard in its environment. The persistence of a chemical contaminant depends on its physico-chemical properties as well as the characteristics of the environment e.g. (temperature) and the biomass material (i.e. the soil or manure composition)^13^. A measure for the chemical’s persistence is the reduction in its concentration over time, for example expressed as the half-life (DT50): the time needed to halve the concentration. In case of a microbiological hazard, the persistence can be reflected by the decrease of the presence of the micro-organism in the environment, which will depend on characteristics related to the micro-organisms themselves as well as environmental conditions, such as temperature or pH.

3. Transfer to the edible parts of the plants or animals

In previous research, various criteria have been identified that can be used to quantify the possible transfer of hazards from one compartment to the other, e.g. from manure to soil, from soil to the plant, from the plant to the animal. Transfer to edible parts of the animal, like meat and eggs, can be expressed using the concentration ratio (concentration in the edible part of the animal divided by the concentration in feed^52^), the transfer factor (concentration of the contaminant in animal products (mg/kg) divided by the daily intake via animal feed (mg/day)^53^) or the bioconcentration factor (BCF) or bioaccumulation factor (BAF) in which the concentration in the animal is divided by the concentration in the feed or the environment^54^.

The transfer of the contaminant from the environment to the plant is influenced by many different factors. For simplicity reasons, we use the mobility of the substance as a measure for this transfer. Substances that are water soluble are mobile and can easily leach to the ground or surface water or can readily be taken up by the plant. The organic carbon-water partition coefficient (*K*_OC_) can be used to express this mobility^13^.

Often data and information on these three indicators (occurrence of the hazards in raw materials, persistence and transfer or accumulation) is lacking and these can only be semi-quantitatively assessed, in terms of low, medium and high. If data are available on one more of them, these needs then be transferred into the three categories. Second, the three indicators that determine the presence of the hazards in foods need to be considered together to arrive at an indication, in terms of low, medium or high, of this presence. Table 9 presents a basis for such a combination of the three indicators to estimate hazard presence.

**Table 9:** Classification of presence and severity of food safety hazards

Occurrence in raw material	Persistence of hazard in environment	Transfer to edible parts of plants/animals	Accumulation	Presence in final product
L	L	L/M/H	–	L
L	M	L	–	L
L	M	M/H	–	M
L	H	L/M/H	–	M
M	L/M	L/M/H	–	M
M	H	L/M/H	–	H
H	L	L/M/H	–	M
H	M	L	–	M
H	M	M/H	–	H
H	H	L/M/H	–	H
L	N/A	N/A	L	L
L	N/A	N/A	M/H	M
M	N/A	N/A	L/M	M
M	N/A	N/A	H	H
H	N/A	N/A	L/M	M
H	N/A	N/A	H	H

Presence in final product	Health effects	Prioritization
L	L/M	L
L	H	M
M	L	L
M	M	M
M	H	H
H	L	M
H	M/H	H

#### Estimating the health effect

Once the presence of food safety hazards in the final food product is assessed using the criteria indicated above, its severity should be taken into account in order to estimate the potential effects on human health. The health effects of a chemical food safety hazard can be assessed by taking into account the four endpoints of toxicity as established by^55^: carcinogenicity (categories Carc.1 A, 1B, 2 according to IARC), mutagenicity (categories Muta.1 A, 1B, 2), reprotoxicity (categories Repr.1 A, 1B, 2), and repeated dose toxicity (category STOT RE 1 and 2). A classification in one of these endpoints is seen as indication of human health risks and is evaluated as high effect on human health^56^. Additionally, health-based guidance values for chronic exposure of human to contaminants via food consumption can be used for classification. Analogous to a previous risk ranking study^24^, chemical hazards can be classified based on their acceptable or tolerable daily intake (ADI or TDI). An ADI/TDI > 10 µg/kg bw/day is seen as having a low human health effect, and an ADI/TDI < = 10 µg/kg bw/day as high human health effect.

For microbial food safety hazards, a similar approach can be followed assigning scores to the pathogens depending on their human health effect. For this purpose, the individual disease burden could be used, which is expressed in Disability Adjusted Life Years (DALYs) per case. The following cut-off values were used in our study: > 0.1 DALY/case, the hazard is seen as posing a high effect on human health, between 0.01 and 0.1 DALY/case as medium effect, and <0.01 DALY/case as a low effect on human health. Based on Bouwknegt, Mangen^57^ this would result in a classification per pathogen as indicated in Table 4.

#### Prioritization

Once the presence of the food safety hazards in the final food products, and the human health effects have been estimated, in a semi-quantitative way, they need to be considered together to arrive at a final risk score which enables the prioritization of the hazards in circular food production. Table 9 presents a starting point for such a combination of presence and health effects scores. In this semi-quantitative assessment we use three levels (low, medium, high) to score each of the parameters such as presence of hazards in foods, health effects and the final risk score. Even though the real life situation is much more complex, data and information on many of the underlying indicators are lacking. Considering three different classes enables distinction in a situation with such a lack of data and information. Overall, the prioritised (high-risk score) hazards will provide risk managers with information on those hazards to focus on first.

Uncertainty can be considers as the change in the overall score in case one of the underlying indicator scores would change. For instance, what would happen with the risk score, if the presence of the hazards moves from medium to high. This can be read from Table 9.

### Application to case studies

Indicators to be used to classify the presence of food safety hazards in food products produced in the circular food system transition are case dependent and do include one or more of the elements: occurrence, persistence and transfer. Note that the severity or health effect of a hazard is independent of the case, as it relates to the food safety hazard in question. The developed framework, as described in the previous section, was applied to three case studies to evaluate the feasibility of the framework, which is elaborated upon below. The case studies were chosen such that they represent typical approaches for circular food production, following the Green Deal, represent a variety of approaches, and were based on data availability.

### Reporting summary

Further information on research design is available in the Nature Research Reporting Summary linked to this article.

### Supplementary information

Reporting Summary

## Data Availability

This study only used secondary data. All data used are presented (or referred to) in the manuscript

## References

[1] De Boer, I. J. M. & Van Ittersum, M. K. *Circularity in Agriculture*. pp. 74 (Wageningen University & Research, 2018).

[2] European Commission, *Communication From The Commission To The European Parliament, The Council, The European Economic And Social Committee And The Committe Of The Regions - A Farm To Fork Strategy For A Fair, Healthy And Environmentally-friendly Food System*. p. 23 (2020).

[3] Jurgilevich A (2016). Transition towards Circular Economy in the Food System. Sustainability.

[4] Gustavsson, J. et al. *Global Food Losses and Food Waste—Extent, Causes and Prevention*. p. 24 (2011).

[5] Van Zanten HHE, Van Ittersum MK, De Boer IJM (2019). The role of farm animals in a circular food system. Glob. Food Security.

[6] zu Ermgassen EKHJ (2016). Reducing the land use of EU pork production: where there’s swill, there’s a way. Food Policy.

[7] Lavelli V (2021). Circular food supply chains – Impact on value addition and safety. Trends Food Sci. Technol..

[8] Focker, M. et al. *Review of Food Safety Hazards in Circular Food Systems in Europe*. Food Research International. p. 111505 (2022). 10.1016/j.foodres.2022.111505 35840214

[9] Goss, M. J., Tubeileh, A. & Goorahoo, D. *Advances in Agronomy* (ed. D. L. Sparks). p. 275-379 (Academic Press, 2013).

[10] Fangueiro D, Alvarenga P, Fragoso R (2021). Horticulture and orchards as new markets for manure valorisation with less environmental impacts. Sustainability.

[11] Berendsen BJA (2018). The persistence of a broad range of antibiotics during calve, pig and broiler manure storage. Chemosphere.

[12] Ghirardini A, Grillini V, Verlicchi P (2020). A review of the occurrence of selected micropollutants and microorganisms in different raw and treated manure – environmental risk due to antibiotics after application to soil. Sci. Total Environ..

[13] Berendsen BJA (2021). A strategy to determine the fate of active chemical compounds in soil; applied to antimicrobially active substances. Chemosphere.

[14] Lahr, J. et al. A step towards the environmental prioritisation of veterinary medicines from animal manurep. p. 8-11 (Knowledge Journal for Water Professionals, 2017).

[15] Pan M, Chu LM (2017). Transfer of antibiotics from wastewater or animal manure to soil and edible crops. Environ. Pollut..

[16] Miller SA, Ferreira JP, LeJeune JT (2022). Antimicrobial use and resistance in plant agriculture: a one health perspective. Agriculture.

[17] Franz E (2007). Quantification of contamination of lettuce by GFP-expressing Escherichia coli O157:H7 and Salmonella enterica serovar Typhimurium. Food Microbiol..

[18] Franz E, Semenov AV, Van Bruggen AHC (2008). Modelling the contamination of lettuce with Escherichia coli O157:H7 from manure-amended soil and the effect of intervention strategies. J. Appl. Microbiol..

[19] Berendsen BJA (2015). The analysis of animal faeces as a tool to monitor antibiotic usage. Talanta.

[20] Tran DTQ (2020). Environmental drivers for persistence of escherichia coli and salmonella in manure-amended soils: a meta-analysis. J. Food Prot..

[21] GLOBAL GAP. *Integrated Farm Assurance Standard with the FSMA Produce Safety Rule Add-on and the**Food Safety and Modernization Act Produce Safety Rule - Comparison Analysis and Guidance* (2019).

[22] Luna-Guevara JJ (2019). The role of pathogenic *E. coli* in fresh vegetables: behavior, contamination factors, and preventive measures. Int. J. Microbiol..

[23] van Overbeek L (2021). Transmission of *Escherichia coli* from manure to root zones of field-grown lettuce and leek plants. Microorganisms.

[24] van Asselt ED (2013). Risk ranking of chemical hazards in food—a case study on antibiotics in the Netherlands. Food Res. Int..

[25] Bosch G (2019). Conversion of organic resources by black soldier fly larvae: Legislation, efficiency and environmental impact. J. Clean. Prod..

[26] Smetana S (2016). Sustainability of insect use for feed and food: Life Cycle Assessment perspective. J. Clean. Prod..

[27] EFSA (2015). Risk profile related to production and consumption of insects as food and feed. EFSA J..

[28] van der Fels-Klerx HJ (2020). Chemical food safety of using former foodstuffs for rearing black soldier fly larvae (Hermetia illucens) for feed and food use. J. Insects Food Feed.

[29] Meyer A (2021). Chemical food safety hazards of insects reared for food and feed. J. Insects Food Feed.

[30] Combalbert S (2012). Fate of steroid hormones and endocrine activities in swine manure disposal and treatment facilities. Water Res..

[31] Cai M (2018). Systematic characterization and proposed pathway of tetracycline degradation in solid waste treatment by Hermetia illucens with intestinal microbiota. Environ. Pollut..

[32] Meijer N (2021). Effects of insecticides on mortality, growth and bioaccumulation in black soldier fly (Hermetia illucens) larvae. PLoS ONE.

[33] EFSA (2018). Risk for animal and human health related to the presenceof dioxins and dioxin-like PCBs in feed and food. EFSA J..

[34] EFSA (2009). Cadmium in food - scientific opinion of the panel on contaminants in the food chain. EFSA J..

[35] EFSA (2012). Lead dietary exposure in the European population. EFSA J..

[36] JECFA. *Oxytetracycline* (WHO, 2002).

[37] EFSA (2020). Scientific opinion–risk assessment of aflatoxins in food. EFSA J..

[38] EFSA (2017). Risks to human and animal health related to the presence of deoxynivalenol and its acetylated and modified forms in food and feed. EFSA J..

[39] Jarzębowski S, Bourlakis M, Bezat-Jarzębowska A (2020). Short food supply chains (SFSC) as local and sustainable systems. Sustainability.

[40] Venema, G. et al. Agrarische productie ten behoeve van de korte ketens. *Een landelijke meting. Wageningen, Wageningen Economic Research.* Report 2021-067 (2021).

[41] Van der Fels-Klerx HJ (2021). Mycotoxins in wheat cultivated in the Netherlands: results from eight years of field surveys. Mycotoxin Res..

[42] Van der Fels-Klerx H (2017). A model for risk-based monitoring of contaminants in feed ingredients. Food Control.

[43] Schaarschmidt S, Fauhl‐Hassek C (2018). The fate of mycotoxins during the processing of wheat for human consumption. Compr. Rev. Food Sci. Food Saf..

[44] EFSA (2016). Appropriateness to set a group health‐based guidance value for zearalenone and its modified forms. EFSA J..

[45] EFSA (2017). Appropriateness to set a group health based guidance value for T2 and HT 2 toxin and its modified forms. EFSA J..

[46] Van der Fels-Klerx HJ (2018). Critical review of methods for risk ranking of food-related hazards, based on risks for human health. Crit. Rev. Food Sci. Nutr..

[47] van Asselt ED (2022). Chemical food safety hazards in circular food systems: a review. Crit. Rev. Food Sci. Nutr..

[48] Chitescu CL, Nicolau AI, Stolker AAM (2013). Uptake of oxytetracycline, sulfamethoxazole and ketoconazole from fertilised soils by plants. Food Addit. Contam.: Part A.

[49] Costello, M. C. S. & L. S. Lee, *Sources, Fate, and Plant Uptake in Agricultural Systems of Per- and Polyfluoroalkyl Substances*. Current Pollution Reports (2020).

[50] Gworek B (2021). Pharmaceuticals in the soil and plant environment: a review. Water Air Soil Pollut..

[51] van der Fels-Klerx, H. J. et al. *Critical Review of Methodology and Application of Risk Ranking for Prioritisation of Food and Feed Related Issues, on the Basis of the Size and Anticipated Health Impact*. EFSA. pp. 210 (2015).

[52] Kan CA, Meijer GAL (2007). The risk of contamination of food with toxic substances present in animal feed. Anim. Feed Sci. Technol..

[53] Leeman WR, Van Den Berg KJ, Houben GF (2007). Transfer of chemicals from feed to animal products: The use of transfer factors in risk assessment. Food Addit. Contam..

[54] Costanza J (2012). Use of the bioaccumulation factor to screen chemicals for bioaccumulation potential. Environ. Toxicol. Chem..

[55] Oltmanns J (2020). Potential emerging chemical risks in the food chain associated with substances registered under REACH. Environ. Sci.: Process. Impacts.

[56] Oltmanns, J. et al. *Final report: Applying A Tested Procedure For The Identification Of Potential Emerging Chemical Risks In The Food Chain To The Substances Registered Under REACH – REACH 2*. *External Scientific Report*. 2019. p. 263 (EFSA, 2019).

[57] Bouwknegt, M. et al. *Disease Burden Of Food-related Pathogens In The Netherlands, 2012*. p. 44. RIVM (Bilthoven, the Netherlands, 2013). 10.1016/j.ijfoodmicro.2014.11.022 25528537

